# Transient common peroneal nerve palsy following skeletal tibial traction in a morbidly obese patient - case report of a preventable complication

**DOI:** 10.1186/1754-9493-6-4

**Published:** 2012-02-21

**Authors:** Frank A Liporace, Richard S Yoon, Anil K Kesani

**Affiliations:** 1 Division of Orthopaedic Trauma, Department of Orthopaedic Trauma, UMDNJ - New Jersey Medical School, Newark NJ 07101, USA; 2 NYU - Hospital for Joint Diseases, Department of Orthopaedic Surgery, New York NY 10003, USA; 3 Division of Orthopaedic Trauma, Department of Orthopaedic Surgery, UMDNJ - New Jersey Medical School, 90 Bergen Street, Suite 1200, Newark NJ 07101, USA

**Keywords:** Skeletal traction, Nerve palsy, Obesity, Lower extremity trauma

## Abstract

Today, skeletal tibial traction remains a mainstay of initial management following high-energy, major orthopaedic lower extremity trauma. Historically utilized as definitive fracture management, recent advances in surgical technology have moved skeletal tibial traction into the realm of temporary management, with benefits including fracture reduction, pain relief, and restoration of disturbed surrounding soft tissues, lowering wound complication and compartment syndrome rates. However, no procedure is without its risks. Here, we present a case of common peroneal palsy following skeletal tibial traction placement, which resolved with subsequent pin removal. Indications, proper placement, potential etiologies, and a review of the literature are also discussed.

## Background

Historically, skeletal tibial traction had been used to definitively treat closed femur fractures [1]. However, as newer surgical options have been developed, skeletal tibial traction for definitive treatment of femur fractures has fallen out of favor for all but the medically debilitated and children below 6 years of age. Rather, the use of skeletal tibial traction as a temporizing measure in major trauma centers has increased [2].

Common indications for the use of skeletal tibial traction include intertrochanteric femur fractures, subtrochanteric femur fractures, femoral shaft fractures, and length unstable distal femur fractures [2]. The goal of traction is to restore the original soft tissue envelope length, by restoring the original bony length to minimize bayoneting as an objective radiological guide [2]. Benefits of skeletal traction include reduction assistance during definitive fixation, reduced incidence of malunion, and avoidance of thigh compartment syndrome [3].

Due to the high incidence of ipsilateral tibial fractures and knee injuries in patients with femoral shaft fractures, as well as the disastrous consequences of placing a tibial traction pin across incidental tumors in the tibial plateau; it is of vital importance that prior to insertion of a tibial traction pin these entities be ruled out with radiographs [4-6]. If uncertainty still exists, more advanced imaging modalities like computer tomographic (CT) and magnetic resonance imaging (MRI) must be obtained. MRI is especially good at identifying ligamentous knee injuries in cases that physical examination proves inconclusive, and should be considered as reported rates of ligamentous knee injuries associated with femur fractures are up to 33% [7].

In addition, one must be aware of the local anatomy neighboring the site for tibial traction pin insertion. Structures at highest risk for injury include the peroneal nerve, anterior tibial artery, and proximal tibial physis in skeletally immature patients. It is important to remember that the peroneal nerve and anterior tibial artery are located posterolateral and posterior to the tibia respectively, at the proximal third of the leg. The course of the anterior tibial artery starts at the bifurcation of the popliteal artery, which is at the lower border of the popliteus muscle, passing forward between the two heads of the tibialis posterior and through the upper border of the interosseous membrane and into the deep part of the anterior leg. Here, it lies close to the medial side of the fibular neck, descending onto the anterior interossesous membrane. Approaching the tibia, the artery lies on the anterior aspect, becoming the dorsalis pedis as it becomes more superficial at the level of the ankle. Moskovich verified the peroneal nerve being posterior to the tibia in the proximal third of the leg during an anatomic study utilizing 6 cadaveric leg dissections, and recommended pin placement through the middle of the tibia in the sagittal plane to avoid neural damage [8].

In a recent study by Rubel et. al, the authors recommended the use of Gerdy's safe zone for invasive intervention to the proximal tibia in order to avoid injury to neurovascular structures [9]. Gerdy's zone was delineated by drawing a circle centered on Gerdy's tubercle with a radius of 45.32 ± 2.6 mm. A line was then dropped from the center of this circle to the head of the fibula. An arc of approximately 100 degrees resulted by connecting the fibular head with the anterior tibial crest, creating a band like "safe" zone.

**Figure 1:**
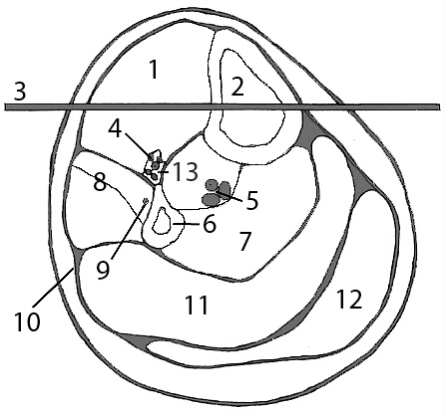
**Schematic representation of the surrounding anatomic structures adjacent to a typically placed tibial traction pin (3)**. 1 - Tibialis anterior, 2 - Tibia, 3 - Traction pin, 4 - Deep peroneal nevrve, 5 - Posterior tibial artery, vein and nerve, 6 - Fibula, 7 - Deep posterior compartment, 8 - Peroneus longus and brevis, 9 - Superficial peroneal nerve, 10 - Deep fascia of leg, 11 - Soleus, 12 - Gastrocnemius, 13 - Anterior tibial vessels.

We recommend insertion of tibial traction pins into Gerdy's safe zone under local anesthesia from lateral to medial in the leg to minimize damage to neurovascular structures. A 7 mm longitudinal skin incision is made 2 cm distal, and 2 cm lateral to the tibial tubercle staying anterior to the fibular head. If inserting a tibial traction pin in a skeletally immature individual, we recommend more distal placement to avoid damage to the proximal tibial and tibial tubercle physis. Use of fluoroscopic imaging can be used to if uncertainty exists. The underlying subcutaneous tissue, and muscle should then be bluntly dissected in line with the incision with a straight clamp. A large threaded Steinman pin or Kirschner wire is then drilled across the tibia in a lateral to medial fashion after centering the pin or wire midway from the anterior and posterior borders of the tibia (Figure 1). The Steinman pin or K-wire is then secured to a traction bow. Application of weights should be applied to the traction pin initially, followed by the thigh and calf via felt pads. Typically, 25 pounds or 17% of body weight is initially applied to the tibial traction pin, and no more than 10 pounds of weight should be applied to the thigh and calf to prevent pressure necrosis of the skin under these pads [10,11]. Clinical judgment, and more importantly traction radiographs should dictate further increments in traction on the pin. Regular monitoring of the skin under the felt pads should be undertaken to identify at risk areas and prevent pressure necrosis.

Traditionally, the application of skeletal tibial traction has been considered a safe procedure with minimal if any complications [12]. Stated benefits include reduction of fracture, and restoration of soft tissue envelope length, making definitive fixation less prone to complications including malreduction and compartment syndrome [13,14]. However, review of the literature demonstrates there are significant complications associated with skeletal tibial traction including pain, pin sites fractures, pin track infections/osteomyelitis, knee stiffness/subluxation, compartment syndrome, and genu recuvatum [10,15-19].

We report an unusual case of a transient common peroneal nerve palsy associated with application of skeletal tibial traction to a distal femur fracture. Our review of the literature has demonstrated only two previous reported cases of transient common peroneal palsy associated with lower extremity traction. However, in both of these instances, emergency technicians applied temporary skin traction in the field using either Sager or Thomas splints [20]. The authors reported that both these cases had incompetence of the lateral collateral ligaments [20]. To the best of our knowledge there have been no documented reports in the literature of transient common peroneal nerve palsy resulting from skeletal tibial traction. Potential risk factors and recommendations to avoid skeletal tibial traction are discussed. In addition, a brief overview of the complications associated with skeletal tibial traction, indications, technique of application, and relevant clinical anatomy are also described.

## Case presentation

**Figure 2:**
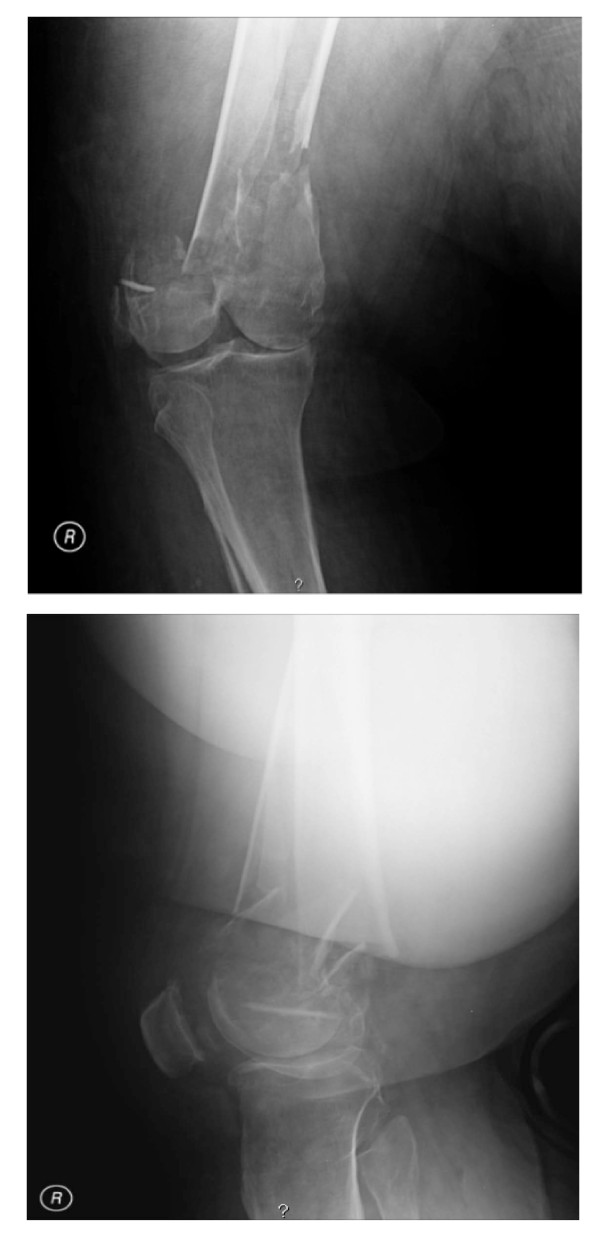
**A-B Pretraction radiograph demonstrating distal femur fracture**.

A 51-year-old, 650 pound, morbidly obese male sustained a comminuted shortened right distal femoral fracture after a motor vehicle accident (Figure 2). On initial examination, he was noted to be neurologically intact in the right lower extremity including his common peroneal nerve distribution. However, due to the body habitus of the patient complete ligamentous evaluation of the knee was not possible either with clinical examination or magnetic resonance imaging.

**Figure 3:**
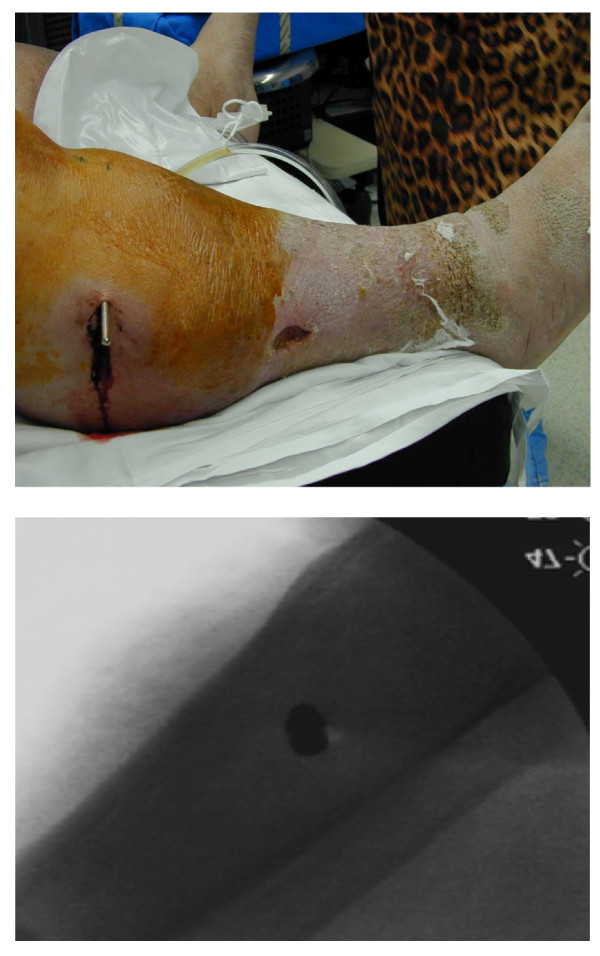
**A-B Picture and radiograph of lower extremity with skeletal tibial traction pin in place**.

Under conscious sedation, he was placed in skeletal tibial traction with the aid of a mini-fluoroscopy device. Subsequent fluoroscopic images revealed the pin to be in the centre of the tibia on lateral imaging (Figure 3).

**Figure 4:**
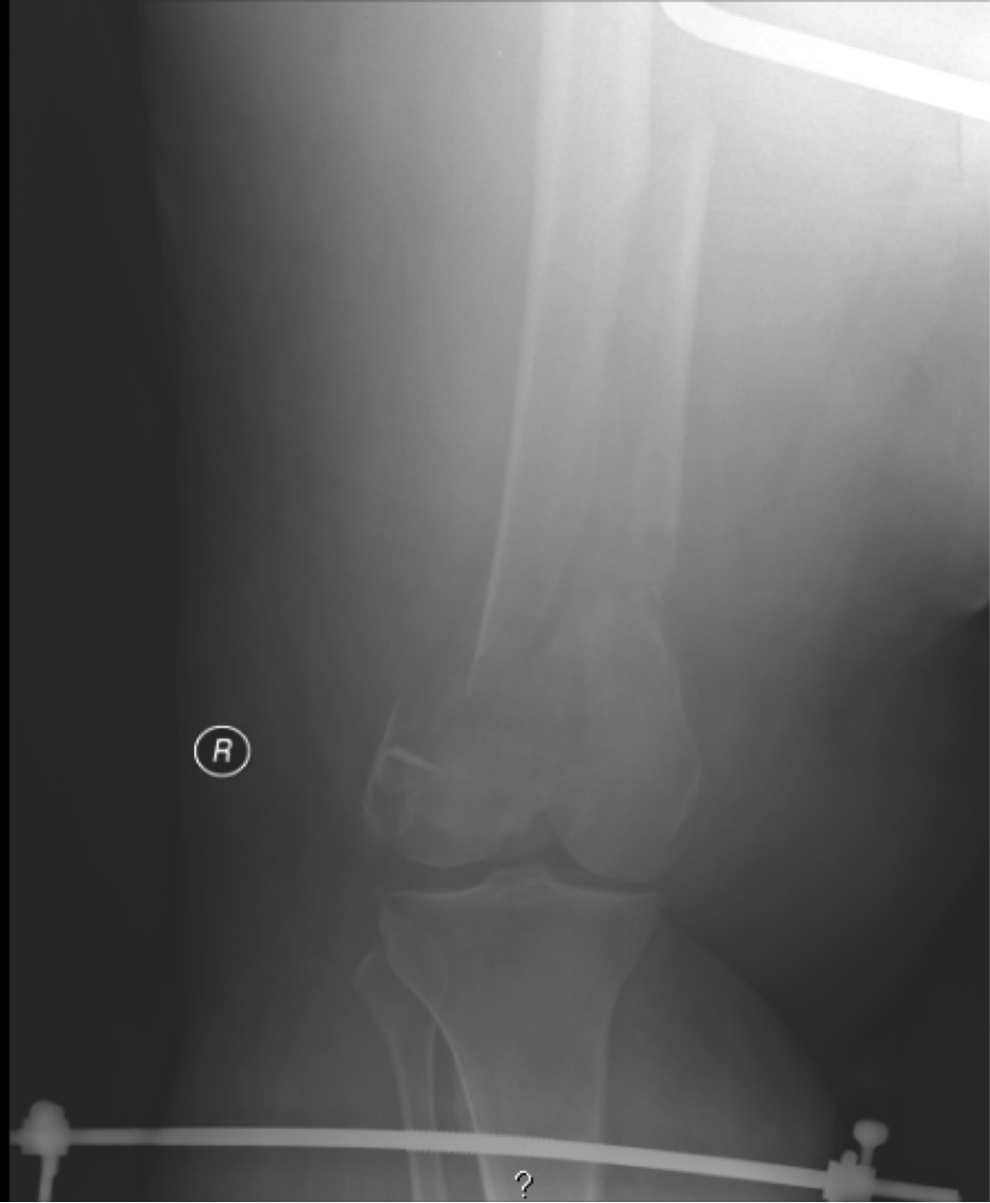
**Posttraction radiograph of distal femur fracture with varus alignment**.

Initially, 25 pounds were applied without any clinical evidence of traction. Sequentially 5 pounds increments were added until the leg had signs of traction being applied to the extremity. A total of 60 pounds traction was applied, however follow-up radiographs still revealed the distal femur to be considerably shortened and impacted (Figure 4).

On follow-up examination of the patient he was noted to have developed a foot drop with loss of sensation over the dorsum of the foot along with the first dorsal webspace. The traction was relieved and he was later placed in a bridging external fixator. Over the next 2 days he recovered complete motor and sensory function in the common peroneal nerve distribution. The patient received definitive ORIF. At 3 years follow-up, the patient remains ambulatory with full motor and sensory distribution of his injured extremity; range of motion remains limited at 0-70, secondary to body habitus.

## Discussion & conclusion

Common peroneal nerve palsy is a potentially devastating complication of skeletal tibial traction, being manifested as pain, numbness, tingling, and in more serious cases, a loss of foot dorsiflexion and eversion. Ultimately, this can lead to decreased function of the lower limb, gait abnormalities, possible loss of ambulation, and an overall decrease in functioning [21]. Current methods of treatment of common peroneal nerve palsy include use of an AFO, muscle transfers and nerve graft procedures [22,23]. However, these treatments are frequently functionally unsatisfactory or aesthetically displeasing for the patient. Hence, prevention of common peroneal nerve palsy is of vital importance.

The peroneal nerve descends in the posterior compartment of the thigh, courses through the popliteal fossa, exits the fossa laterally, passes over the posterior aspect of the fibular head and then winds around the fibular neck in a very superficial position leaving it vulnerable to injury on the lateral aspect of the knee.

In our case, we believe one possible etiology for the common peroneal nerve injury may have been unrecognized ligamentous injury to the lateral collateral ligament allowing a varus deformity to occur once traction was applied. In turn, leading to widening of the lateral joint space and a concomitant stretching of the common peroneal nerve. Similarly, another possible etiology in our case was a lateral femoral condyle fracture that potentially functioned as a complete lateral collateral ligament injury due to the lack of proximal attachment of the ligament to a fixed point. An additional etiology that may have contributed to transient common peroneal injury in our case is hyperextension at the distal femoral fracture site with traction, leading to compression of the peroneal division of the sciatic nerve by the fracture fragments.

Hence, it is imperative that a careful physical examination for integrity of lateral collateral ligament be performed, as well as examination of the fracture pattern prior to application of skeletal tibial traction. Injury to the lateral collateral ligament is a contraindication to tibial traction. However, in our case the body habitus precluded a thorough ligamentous evaluation. Therefore, in situations where one suspects injury to lateral collateral ligament, but is unable to definitively determine the absence of liagmentous injury, one should consider it as a relative contraindication for traction. In such a situation, a safer option would be to temporize the limb in a transarticular external fixator. External fixation allows application of traction in a more controlled fashion. One can apply traction with the knee in neutral, or even slight valgus to prevent stretching of the common peroneal nerve. Furthermore, traction can be applied with the knee in slight flexion to avoid extension at the fracture site and minimize damage to popliteal fossa structures.

Another temporizing option applicable in certain situations is distal femoral skeletal traction, such as in cases when the distal femoral fracture is extraarticular and proximal enough to produce a large distal fragment. Although, one must be aware that pin placement here can potentially contaminate or complicate later internal fixation.

Review of the literature demonstrates that human nerves can withstand an increase in length of 4-11% before histological structural compromise occurs [24-26]. Further stretching of the nerve beyond 8% can lead to reduced perfusion by the microcirculation by narrowing the intraneural and extraneural microvasculature, initially resulting in a reversible loss in function [27]. If allowed to continue, prolonged ischemia could lead to a more permanent form of injury. Hence, early detection of peroneal nerve palsy with frequent neurovascular examinations especially when increasing traction loads is of vital importance, and rapid removal of traction as in our case may lead to full recovery of sensory and motor function.

In conclusion, complications associated with placement of lower extremity in skeletal tibial traction are rare, but potentially devastating. Hence, prevention of iatrogenic traction induced common peroneal nerve palsy by a thorough assessment of the integrity of the knee lateral collateral ligament complex is important prior to application of skeletal tibial traction. Furthermore, once traction is applied, careful monitoring of the neurovascular status of the limb particularly when increasing traction loads must be undertaken to allow rapid removal of traction to avoid irreversible nerve damage and prevent poorer outcomes.

## Consent

Written informed consent was obtained from the patient for publication of this Case report and any accompanying images. A copy of the written consent is available for review by the Editor-in-Chief of this journal

## Abbreviations

CT: computed tomography; MRI: Magnetic resonance imaging; K-wire: Kirschner wire; ORIF: Open reduction internal fixation; AFO: Ankle foot orthosis

## Competing interests

The authors declare that they have no competing interests.

## Authors' contributions

FAL Conceived design, patient care, structure, final editing and approval, RSY Conceived design, patient care, data collection, first draft, final editing and approval, AKK First draft completion, patient care, final editing and approval. All authors read and approved the final manuscript.
